# University Social Responsibility, Service Learning, and Students' Personal, Professional, and Civic Education

**DOI:** 10.3389/fpsyg.2021.617300

**Published:** 2021-02-25

**Authors:** Márcia Coelho, Isabel Menezes

**Affiliations:** Centre for Research and Intervention in Education (CIIE), Faculty of Psychology and Education Sciences, University of Porto, Porto, Portugal

**Keywords:** University social responsibility, higher education, service learning, students, transversal competences, experiential learning

## Abstract

The long-standing vision of universities as the “*alma mater*” of students and graduates is a demonstration of its role as sustaining the person, the expert/professional, and the citizen. This role has persisted in the face of rising global challenges such as the emergence of new learning spaces, the growing diversity of publics, the call for productivity and performativity, and the hope for a significant engagement with the community and the public good. These sometimes conflicting tendencies have also stimulated higher education institutions to further pedagogical strategies that articulate in novel ways the classical elements of learning: action/experience and reflection/theory. In this context, service learning received a new impetus, particularly in the post-Bologna European Higher Education Area, as universities were looking for ways in which to articulate the social dimension of HEI and their “third mission” as institutions not only committed to addressing and solving societal problems, but also committed to fostering public-minded alumni through powerful experiences of engagement for both the students and the community.

This paper is based on the experience of Erasmus+project ESSA, a service earning based project focused on University social responsibility (USR). ESSA engaged four groups of students from three European universities (Edinburgh, Porto, and Kaunas) in conducting a 1-week on-site USR audit based on an ecologic and situated concept of social responsibility. We will consider the perceived impact of ESSA on 44 students through a thematic analysis of focus group discussions and student self-assessment reports produced during and after their participation in the USR audit.

## Introduction

University social responsibility (USR) has gained momentum in the European higher education context. Facing the ascent of global challenges led by new learning spaces and working methods, the climate crisis, the large scale population movements, and the growing diversity of publics, higher education institutions (HEIs) are set with new demands both in the sense of rethinking their internal practices as well as their interaction with the community, balancing tensions between inclusion and quality. If, on one hand, HEIs are pressed to remain competitive and respond effectively to the different challenges of globalization (Zgaga, 2019), on the other hand, they also need to prepare students for their future professional activities as well as for their lives as critical and engaged citizens. Recent phenomena involving the pandemics, the rise of populism and the menace of fake news have come to demonstrate not only the significance, but even the urgency of the latter.

The articulation between the University and its social role is “extremely important, particularly when universities are not only the most enduring institutions of civilization but also because they are now becoming a global knowledge industry” (Shek et al., 2017, 25). However, the challenge University is facing is not confined exclusively to its capacity to produce knowledge, but also to its potential to link it with citizenship, in such a way that its role is also important when it comes to structuring the social and cultural dimension of the knowledge society (Delanty, 2009).

In fact, there is a growing recognition and call for the social/public responsibility of HE, urging HEI to identify and address issues that affect the well-being of communities, nations, and global society and to introduce a range of innovative educational methods capable of fostering students' critical thinking and creativity within but also beyond subject knowledge (Santos et al., 2016). That requires students to fully develop their own abilities with a sense of social responsibility, educating them to become critical participants in a democratic society and promoters of changes that will foster equity and justice (UNESCO, 1998; Simons and Masschelein, 2009). Adding to that, on the post-Bologna European context, special emphasis was placed on providing equal opportunities for all in terms of access, progress and completion of studies, despite different cultural, and social backgrounds (European Ministers in charge of Higher Education, 2007).

These imperative demands have subsequently been taken to the next level in the United Nations's Sustainable Development Goals (United Nations, 2015), which aim that, by 2030, all learners acquire the knowledge and the skills needed to promote sustainable development, including, among others, human rights, gender equality, promotion of a culture of peace and non-violence, global citizenship, and appreciation of cultural diversity.

However, the concept of USR is elusive, ranging from a continuum between a conservative pole and a critical-transformative pole (Menezes et al., 2018). The conservative pole is based on a perspective of organizational governance, keeping the field of research and teaching untouched, and seeing USR as an opportunity and positioning strategy in the context of the marketization of HE (Velazquez et al., 2006). Within the critical-transformative pole, USR crosses the mission of universities as a whole, involving a deep change at all levels of the institutional endeavor (Vallaeys et al., 2009; Barnett, 2011; Amorim et al., 2015). Situating our approach in this last pole, this implies an ecological-situated vision of USR (Menezes et al., 2018), recognizing the significance of the interaction between the University and its contexts, and allowing for a more grounded view of the role and the commitment of University to the common good. Indeed, University as “a critical and an enquiring University (…) acting to put its resources to good effect in promoting world well-being. It will be active on the local and regional stages and, very often, on the world stage” (Barnett, 2011, 252).

Although there is no consensus on the meanings of USR, it is clear that the role of HEI in the development and transformation of their social environment is crucial (Nunez, 2019). This involves not only the advancement of science and the sharing of knowledge with and for society but also the empowerment of students through the exercise of active, inclusive, participatory, and democratic citizenship.

This vision is aligned with thinking the formative process of students as a continuum that extends throughout life, which supposes both a personal/autonomous and a collaborative/shared quest for knowledge and understanding, that allows one to adapt to an increasingly mutable social space and to mutate it as well, not only as an individual but in conjunction with others. As such, HEI are responsible for promoting multiple learning spaces and need to develop opportunities, in addition to study plans, that allows students to learn in context and in cooperation with others, like USR projects based on service learning (Santos et al., 2016). Linking USR to grounded and reflective action through service learning projects can surely generate significant learning spaces for students, fostering important capacities related to decision-making, problem-solving, research, analysis, and negotiation, that ultimately can allow them to become more aware, collaborative, and creative in their professional activities, remaining alert to the innovations that can involve social and environmental improvements for all (Vallaeys et al., 2009; Lopes, 2015). Resch (2018) underlies that service learning USR projects can potentiate socially responsible students as they feel their impact on services and community by actively participating in relevant socially responsible projects. Moreover, the successful establishment of a service learning culture at the University can develop students' outcomes as their personal and interpersonal achievements, understanding and applying knowledge, engagement, curiosity, reflective practice, perspective transformation, citizenship, and social responsibility.

However, despite the proliferation of research in this area, there is a tendency to focus on the meanings of USR or on identifying benchmarks, while the potential impact of students' involvement in USR projects is not yet sufficiently studied. This is pointed out as a significant literature gap (Larrán et al., 2012) and constitutes the background of this project that has the main objective of producing knowledge about University Social Responsibility in the European context. Our main goal is to explore whether and how USR service learning projects students' development in relation to their academic, civic and professional life, and to advance our knowledge on how these dimensions intersect with each other and with students' vision of the role of universities. Our guiding question is: What are students' perceptions of the potential impact of USR service learning projects on their own academic, civic, and professional development?

## Research Context and Methodology

This research is carried out in the context of the ESSA Project–European Students, Sustainability Auditing (funded by the European Commission's Erasmus+, see essaproject.eu/about), which comprises a consortium of European universities: University of Edinburgh, Kaunas University of Technology, and University of Porto, together with their student associations, the European Student Union, and the National Union of Students of the United Kingdom.

Involving students, teachers, researchers, and staff members as active agents in their universities, this project aimed to develop a certificated programme for students in USR auditing. This innovative project, based on experiential learning and student-centered learning, provides a unique opportunity to complement students' training by acquiring USR auditing skills and other transversal and transferable competences.

The rationale for the student training was a reference framework for USR in Europe (Amorim et al., 2015) based on four benchmarks: 1. Research, Teaching, Support For Learning, and Public Engagement; 2. Governance; 3. Environmental and Societal Sustainability and 4. Fair Practices. The whole project involved two phases. In a first phase, all the selected students attended, in their host institution, an intensive training based on the concepts of USR, auditing, research methods, and techniques. The curricula of this intensive training was conjointly developed by trainers from the three universities during a week-long training at the University of Porto at the outset of the project (Coelho et al., 2019). In a second phase, students conducted one of four supervised audit exercises on a partner University. Working in multicultural groups (seven students from each University in a total of 14 to 20 students for each audit exercise), students produced an audit report based on the qualitative analysis of official documents, reports, and public information, along with interviews and focus groups discussions with universities officials (e.g., vice rectors, deans, senior management, students' associations, unions representatives) and stakeholders (e.g., community organizations, institutional partners). The whole process involved a close collaboration between the student auditors and the local University. Auditing is a process that enables an organization to assess and report its performance, to establish where it is at and to provide critical feedback, ultimately improving its performance and narrowing gaps between vision/goals and reality (Jain and Polman, 2003). However, under ESSA, the auditing was conceived as an ecological process:

“*Producing situated knowledge means taking into account of the various actors' points of view about their experiences—and therefore, including what and how they conceive USR in the analysis and discussion of the audit “results.” It also implies approaching the audit process broadly, intentionally involving disempowered or disenfranchised groups in the University. This approach cannot ignore that there is a potentially oppressive role played by societal and normative structures, that constrain universities in ways that should be acknowledged and included in recommendations for change. Last, but not least, one should recognize the limits of our endeavor: as “the map is not the territory,” to quote Borges, the University is not the audit report*” (Coelho et al., 2019, 33).

As such, the auditing exercise is not only a highly challenging and hands-on learning, but it includes the elements commonly recognized as essential to service learning: experientially based approach; learning focus on behavior, field-based learning, and on reflection (Witmer and Anderson, 1994).

Although ESSA is not a completely typical service learning project, in particular when it comes to working “in and with communities,” it is important to keep in mind that universities are (also) communities, and ESSA student auditors worked in and with universities to improve their social responsibility, with goals of civic learning being at the core of both individual students' and organizational learning. In this sense, ESSA is quite aligned with Bringle and Hatcher (1999, 180) definition: “a course-based, credit-bearing educational experience in which students (a) participate in an organized service activity that meets identified community needs and (b) reflect on the service activity in such a way as to gain further understanding of course content, a broader appreciation of the discipline, and an enhanced sense of personal values and civic responsibility.”

The goal was to develop students' personal, civic, and political competences as critical agents of social responsibility, involving them, in a more committed and profound way, with their universities and, more broadly, with higher education, hoping that this experience may translate into an increase in their employability and commitment to USR, but also of the quality of the University experience itself, through the contact with different University members and community organizations where the University is involved. In fact, this project is an example that “offers students, faculty, University, and community partners a distinct opportunity for improving their practical and communication skills, enhancing their sense of social responsibility and developing a better understanding of the connection between theory and practice” (Cheng, 2018, 423), and also to strengthen the relations between the University and the community.

This study is exploratory and evaluative in nature as it takes the ESSA project as its context. However, it aims to understand, more broadly, how students perceive the impact of participation in USR projects, in general, in the development of their academic, civic, and professional life. The project uses focus groups discussions to address specifically the experience of 44 students who participated in the ESSA USR audit exercises. Participants involve all the ESSA students who agreed, on a voluntary basis, through a written informed consent, to share their self-assessment reports (SAR) and to participate on focus group discussion conducted immediately after the audits (three focus groups, *n* = 32) or after the completion of the assessment procedure (three focus groups, *n* = 12), in both English and Portuguese. The majority of the participants are female (68%) and the medium age is 22,5 years (SD = 3,4 years); as seen on Table 1 participants' academic background is quite varied. Confidentiality of the participants has been guaranteed and the use of records and data were subject to standard data protection regulations of the Faculty of Psychology and Educational Sciences of the University of Porto. All the focus groups were conducted by the first author and audio recorded, being subsequently transcribed verbatim and stored according to data protection procedures and with the support of NVivo software for data management and analysis.

**Table 1:** Focus groups participants.

		**University**			
		**UP**	**UoE**	**KUT**	**Total**
Gender	Male	7	2	5	14
	Female	11	11	8	30
Area of studies	Sciences and Engineering	4	7	4	15
	Education, human, and social sciences	7	3	4	14
	Medicine	4	0	0	4
	Law and international studies	3	3	5	11
Total		18	13	13	44

*UP, University of Porto; UoE, University of Edinburgh; KUT, Kaunas University of Technology*.

Qualitative thematic analysis was used to analyse data because it is a dynamic and flexible method in the treatment of qualitative data, which allows a more diverse description of the data, focusing on the identification and description of common themes, enhancing understanding of the explicit and implicit meanings associated with the textual data (Braun and Clarke, 2006).

Working with thematic analysis implies a priori decision making on theoretical choices and approaches. Thus, in this research, we have opted for a contextualist approach, “sitting between the two poles of essentialism and constructionism, and characterized by theories such as critical realism which acknowledge the ways individuals make meaning of their experience, and, in turn, the ways the broader social context impinges on those meanings” (Braun and Clarke, 2006, 9). This was particularly adequate as our goal was to understand how students view the potential impact of USR projects on their own development, and how these perceptions interconnect with their vision of the University.

**Figure 1:**
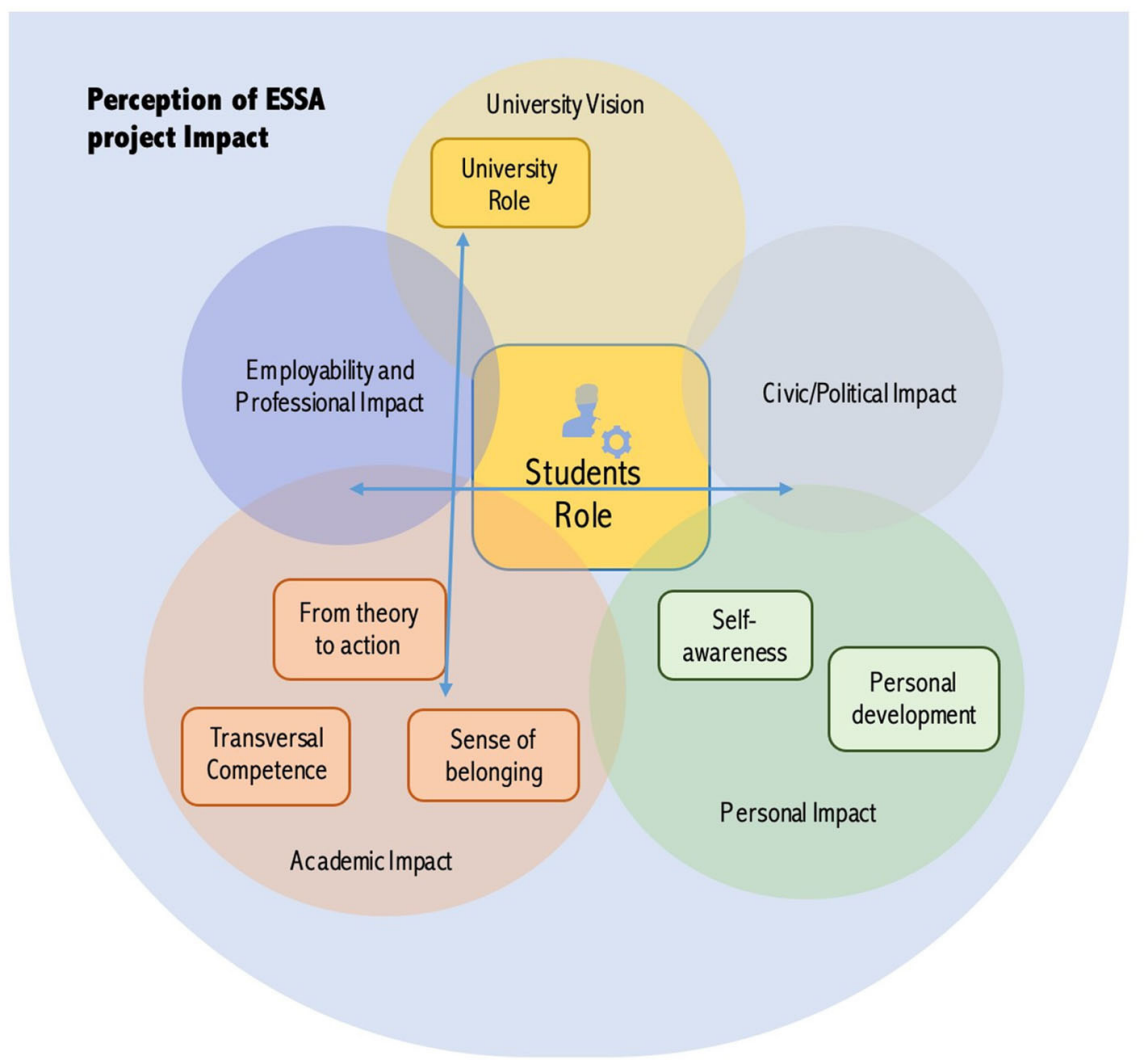
Thematic Map.

Consequently, and according to Braun and Clarke (2006), the themes can result from both an inductive or/and deductive process. In this study, the themes were identified firstly in relation to the theoretical focus of the research and were based on the initial coding process, in a second moment, emerging from the data to allow a deeper understanding of the conceptual relationships established by the students. This means that both semantic and latent approaches sustained the development of the thematic analysis map that depicts “the overall story (…) about the data” (Braun and Clarke, 2006, 21) (see more details in Figure 1). The coding process involved an ongoing discussion and review by the two authors in order to ensure a deeper understanding of the data (Patton, 1999).

This analysis followed Braun and Clarke (2006, 2013) recommendations of six steps to complete the analysis: (1) familiarizing with the data; (2) generating initial codes; (3) searching for themes; (4) reviewing themes; (5) defining and naming themes and (6) producing the report. The subsequent results section will specifically address an overview of the theme “perceived impact of ESSA project” in the different sub themes and codes mapped.

## Results

The preliminary findings suggest that that the ESSA project can be a motor to “empower students as critical agents of social responsibility” (Coelho et al., 2017, 1173), showing that students consider the participation in USR projects as a turning point in their development: “*It was easier to try to say what it was that didn't change me!*” (Female, Medicine, UPorto).

Based on the thematic map as a graphical representation that illustrates the general conceptualization of data patterns and the relationships between them (Braun and Clarke, 2006), the theme “perception of ESSA project impact on students” had a major expression in all the data analyzed and from where derivate five sub themes and seven codes that are interconnected (see more details in Figure 1).

The perception of impact on their **vision of the University** underlies students' perception of impact in two different and interconnected codes: the perception of the **University role** and the **students role** as core sub-themes because they predict what will be the perception of impact on other dimensions. Some students understand the **University role** in a more engaged way with themselves and society:

“*The impact that the University can have on the community and how important it is to bring life to the city! I don't think we can see that yet. The University for me is much more of an engine for growth in the city than tourism, because tourism is temporary, the University is bringing and doing knowledge here*” (FGD3–Female, Medicine, UPorto).

They even understand themselves as part of it: “*the University doesn't do it by itself, if we don't try to shape it, we're not doing much here eithe*r” (FGD2–Male, Law, UPorto), and tend to think of their role as a student in a more active sense, because they are more aware of the initiatives and of the ways the University functions.

On the other hand, they tend to perceive that the University does not involve the majority of students in its core activities and decisions and indicate that this involvement is a turning point. For some of them, the relationship with the University is more distant and based only on a relationship of service provision–just a place to get knowledge and prepare to work:

“*our University has this strategy to be a greener University but the students don't know about it! It's like a strategy in “higher places” and if you ask a normal student they don't know*” (FGD5–Female, European Studies, UEdinburgh), “*no one actually gets them involved*” (FGD6–Female, International Relations, KTU).

Perceptions of students role are therefore linked with the perception of the University role and with students' experience in USR projects. Students who understand the University role as more connected to themselves and society, think of their role as more active not only in the academia, but also in the community:

“*Part of being a student is also thinking about our role in academia. That is one of the reasons that led me to be very involved in students' unions and projects*” (SAR1–Male, Medicine, and Philosophy, UPorto).

There students, even if they comply with their academic demands, they are much more than “just a student:” they participate in projects and students' associations, have an opinion/action regarding important social issues and fight to drive change if needed, many times engaging in several SR initiatives in the University and outside. On a different pole, students who see the University as an isolated and impenetrable institution see themselves as just a client without a real voice, ask for the basics, and even if they can identify problems, they think their opinion is an isolated one. For the majority of these students this was their first participation in SR projects:

“*Before the ESSA project I had only a basic understanding of USR, but training and auditing activities substantially increased my awareness of USR. Now I have the ability to describe and discuss why USR is an important area for any University*” (FGD6–Female, Chemical Engineering, KTU), “*the University I study in (…) students' motivation for sustainable development initiatives is low, participation in international networks is slightly growing and is not improved*” (SAR30–Female, Public Administration, KTU).

Perceptions of students' role are linked with several other sub-themes and codes, predicting impact on other areas. For instance, students' opinions regarding the **impact on their civic and political life** were fairly homogeneous even between students from different nationalities and universities. They highlighted the impact in the growth of their active civic participation and mostly in the knowledge and conscience about social responsibility, the capacity to identify social responsible companies and the need to acknowledge that everything has an impact and that they are obliged to make the difference. Students who perceive their role in a more engaged way tend to be more active in the community and be a part of other social responsibility projects in their communities:

“*in the relationship with the other, in the question of community and society, instead of just being locked into a certain kind of knowledge, with the rest, we seem to be illiterate, it is not, of course, but I think it is a little bit out there*” (SAR5–Female, Psychology, UPorto); “*let's put it this way, if it serves to train more decent citizens it is already worthwhile, regardless of the return it has on employability*” (FGD2–Male, Law, UPorto).

As for the **personal impact**, students have identified two different areas: the impact on their **personal development** and on their **self-awareness** that are strongly connected with the perceived impact on their civic and political life. Students consider that the project has been a very important learning space for their **personal development** in terms of skills related to tolerance and acceptance of others and their views, developing their collaborative profile not only in the University, but in other spheres of their lives:

“*For me it made me think (…) about how I react in certain circumstances, like, for example, I tend to be very impulsive and kind of telling people what to do instead of maybe being more collaborative and negotiate… rather than telling people what to do…*” (FGD5–Female, Geo Sciences, UEdinburgh), “*To do something and additionally, if you don't have a plan while you're doing this kind of projects and pushing you in certain ways which you haven't thought*” (FGD6–Male, Marketing, KTU).

Connected with the perceived personal impact, students also mentioned the opportunity for **self-awareness** that this experience brought, and it was unanimous that it was one of the major advantages of the experience for all the students, with or without previous experience in SR projects:

“*in general this kind of project does give you an insight to what is what you're good at and what you're not, so you should just work on things that you're good at, not try to compensate for what you're not. So you're just wasting your time basically, if you're trying to press yourself just to fit for the moment (…) you recognize your real personality, who you are*” (FGD6–Female, Language and English, UEdinburgh).

The perceptions of **academic impact** include all the references to the impact on students' academic life, such as the development of a **sense of belonging**, acquiring **transversal competences**, and the possibility of moving **from theory to action**. A significant number of students described a deeper **sense of belonging** to the University, linked to an increased institutional involvement and sense of unity that also triggered a new vision of the University:

“*very different contexts, very different people, people from very different faculties, [it] was funny because it triggered in me a sense of unity of the University that I had never felt, for me my University was this faculty, when in fact (…) I realized that sense of unity that was perhaps much more interesting”* (FGD1–Female, Psychology, UPorto).

“*the idea of entering University to be trained to be something very specific, I think it will end up being diluted and offering a much more holistic training, in the sense of the education of the person, of the human development and not only training for that profession*” (FGD4–Male, Sociology, UPorto).

In that sense, they identify the clear impact of their participation in the acquisition of **transversal competences** like leadership, team work, public presentation skills, and time management, but also in how they envision their training and the way they were able to pass **from theory to action**. Students have repeatedly mentioned that the possibility of having a real experience in a real context, even if not in their study field, was very different from the opportunities they had had so far in their studies:

“*I also reckon that, considering my academic area of studies, the conduct of an audit proved very relevant to me, as it put into perspective theoretical knowledge, showing me how it can be practiced, with real people, in real scenarios, and how flexible and resilient we've got to be in this kind of task”* (SAR27–Female, Education Sciences, UPorto).

Even if the competences acquired and developed were not considered the core of their studies and future professional life, students were able to identify them and their importance for them as active and engaged students and citizens.

“*I feel that now when I get to the faculty and I have to stick to those schedules and do certain things with which I don't identify so much, it will be a bit difficult… a contrast to what I have been doing this and I have been learning so much and I fear that when I get there I will be able to apply everything I have learned in this context? Of course, the ability to observe, to think critically, to work as a group, to organize, I don't think there's much of a problem. But to think that when I get there, considering the time I've been to University, I think that maybe it won't be so easy for me to do half of what we did here this week”* (FGD4–Male, Medicine, UPorto).

Linked with **transversal competences** and **from theory to action**, is the impact on their **employability and professional life**. Some students underlie their own development of skills and empowered transversal capacities, hands-on learning and field experience in the area of their studies, and how this might be an advantage in the future:

“*As my future job heavily relies on the work toward the community, especially due to my interest in Public Health, addressing inequalities, ethical problems, and reconsidering the usage or efficiency of resources is an essential pillar of my upcoming profession*” (SAR41–Male, Medicine, UPorto).

Other students assume a more externalized focus, related to the concerns that the labor market may (or may not) have with social responsibility and the appreciation of these skills and concerns:

“*Yes, it makes us more employable. As it is a pilot program, it is a niche as well and people will want to talk about it and that will give us a chance to share the knowledge we gained and talk about the skills we gained”* (SAR18–Female, Social History, UEdinburgh);

“*Yes and I think it's good to share knowledge, I am personally looking to have higher education jobs in the future and I am going to be critical about what I will apply based on things like this! And if like I have an interview for something that I think it is not really for me, I can still talk about this and read the other person or the company, and what they think about it and actually see if it's [“worth a try”]*” (FGD5–Female, Arts, KTU).

## Discussion

Understanding the University as a multiple learning space, its potential impact on students' life paths should not be limited to training them as increasingly specialized professionals, but rather to influence various facets of their personal, social, civic, and professional development, that goes far beyond academic training (Rutti et al., 2016). USR projects, such as ESSA, create opportunities for balancing theory and practice, action, and reflection, in ways that foster students' development and empowerment in different spheres of their life. Our findings suggest that students' involvement in projects such as ESSA can change the way they conceive their academic life, but also their roles in and out of the University, thus furthering “pro-active human minds for the full exercise of citizenship through creative actions capable of building socially responsible and economically sustainable societies” (Ribeiro and Magalhães, 2014, 135).

USR must be at the core of higher education institutions, integrated in all spheres of higher education institutions, from teaching and research, to governance and community engagement (Villa, 2014). This is why service learning USR projects, such as ESSA, can be of significance in the lives of both universities and students, as they guide higher education institutions in fulfilling social responsibility in multiples ways. This happens because these projects reinforce the connection with the community, recognizing that it enables opportunities for significant learning and for putting knowledge in action (Santos et al., 2016). In fact, “learning in service is a pedagogical model that allows the University to exercise its social responsibility in the formative environment through teaching-learning processes linked with its social environment” (Nunez, 2019, 97). By focusing students' learning on real world problem solving, service learning USR projects promote more powerful connections between theory and practice and foster students' transversal and transferable skills, building bridges between the academy and communities.

Our findings suggest that the potential for learning and change is there, at least from the perspective of students. This is, surely, an important limitation of our work, with its sole focus on students' perceptions, along with the potential limitation of the selection bias linked with the fact that ESSA was a volunteer project and the students could/did participate on other projects/activities, already being more connected to USR to begin with. Nevertheless, the consistency of the findings–involving four cohorts of students, from three universities/countries and involved in an USR audit in different points in time–suggests the significance of this service learning USR project. Nevertheless, more research is necessary, particularly following up on if and how these learning experiences are re-signified as students transition to their professional lives and are confronted with other personal, professional, and civic challenges.

## Data Availability Statement

The raw data supporting the conclusions of this article will be made available by the authors, without undue reservation.

## Ethics Statement

The studies involving human participants were reviewed and approved by Programa Doutoral em Ciências da Educação in accordance with Comissão de Ética da Faculdade de Psicologia e de Ciências da Educação. The patients/participants provided their written informed consent to participate in this study. Written informed consent was obtained from the individual(s) for the publication of any potentially identifiable images or data included in this article.

## Author Contributions

MC did the data collection using focus groups and analysis (focus groups and students' reports), as well as most of the writing of the paper. IM designed the study, supervised the research work, regarding the construction of focus group scripts and the discussion of the thematic map, and involved in some of the writing and the final revision of the paper. All authors contributed to the article and approved the submitted version.

## Conflict of Interest

The authors declare that the research was conducted in the absence of any commercial or financial relationships that could be construed as a potential conflict of interest.
